# Mastering your fellowship

**DOI:** 10.4102/safp.v62i1.5141

**Published:** 2020-06-11

**Authors:** Mergan Naidoo, Klaus B. von Pressentin, Andrew Ross, Tasleem Ras

**Affiliations:** 1 Department of Family Medicine, University of KwaZulu-Natal, Durban, South Africa; 2 Division of Family Medicine, University of Cape Town, Cape Town, South Africa

**Keywords:** Fellowship of the College of Family Physicians of South Africa, FCFP (SA) examination, family medicine registrars

## Abstract

The series, ‘Mastering your Fellowship’, provides examples of the question format encountered in the written and clinical examinations, Part A of the Fellowship of the College of Family Physicians of South Africa (FCFP [SA]) examination. The series is aimed at helping family medicine registrars prepare for this examination. Model answers are available online.

## Introduction

This section in the *South African Family Practice* journal is aimed at helping registrars prepare for the Fellowship of the College of Family Physicians (FCFP [SA]) Final Part A examination and will provide examples of the question formats encountered in the written examination: Multiple choice question (MCQ) in the form of single best answer (SBA – Type A) and/or extended matching question (EMQ – Type R); short answer question (SAQ), questions based on the critical reading of a journal (evidence-based medicine) and an example of an Objectively Structured Clinical Examination (OSCE) question. Each of these question types is presented based on the College of Family Physicians’ blueprint and the key learning outcomes of the FCFP programme. The MCQs will be based on the 10 clinical domains of family medicine, the EMQs will be aligned with the five national unit standards; and the critical reading section will include evidence-based medicine and primary care research methods.

This month’s edition is based on Unit Standards 1 (critically reviewing new evidence and applying the evidence in practice), Unit Standards 2 (evaluate and manage a patient according to the bio-psycho-social approach) and Unit Standards 5 (conducting all aspects of healthcare in an ethical and professional manner). The theme for this edition is anaesthesia and critical care and a few of the questions have been aligned with the current coronavirus disease 2019 (COVID-19) pandemic. We suggest that you attempt answering the questions (by yourself or with peers/supervisors), before finding the model answers online at https://www.safpj.co.za/.

Please visit the Colleges of Medicine of South Africa’s website for guidelines on the Fellowship examination at https://www.cmsa.co.za/view_exam.aspx?QualificationID=9.

We are keen to hear about how this series is assisting registrars and their supervisors in preparing for the FCFP (SA) examination. Please email us your feedback and suggestions.

## Multiple choice question: Single best answer

A 25-year-old, 70 kg male with no known comorbidities presents with a cough, fever, difficult breathing and respiratory distress. He is rapidly intubated and placed on a ventilator. His blood pressure = 100/60 mmHg, pulse = 105/minute, respiratory rate = 24/min, temperature = 38 °C and his oxygen saturation = 85% on 100% oxygen. The ventilator mode is assist-control with a set rate of 14 breaths/min (br/min), tidal volume = 420 mL, peak end-expiratory pressure = 6 cm H_2_O and plateau pressure = 21 cm H_2_O. The patient has been accepted at the local intensive care unit, but a delay is anticipated. The most appropriate next step is to:

- Change the ventilator mode to a pressure mode.
- Increase the peak end expiratory pressure to 10 cm H_2_O.
- Increase the set respiratory rate to 16 br/min.
- Increase the tidal volume to 550 mL/kg.
- Sedate the patient to supress the respiratory effort.

*Answer*: (d)

The advent of the COVID-19 pandemic has resulted in many primary care providers needing to gain insight into the critical care management of patients. COVID-19 is the disease caused by severe acute respiratory syndrome coronavirus-2 (SARS-CoV-2) and presents with mild to moderate illness in 80% of patients, severe illness in 15% of patients and critical illness in 5% of patients. The reproduction ratio of the disease and the series interval are alarming and have already had devastating effects on healthcare systems around the world. The anticipated surge in cases in South Africa in the coming months is expected to stress the healthcare system. The management of limited resources, such as intensive care unit beds, is expected to come under severe strain, and plans are in place to improve the capacity at lower levels of care to deal with severely ill patients presenting with severe respiratory distress.

Our knowledge of COVID-19 is evolving, almost daily, and as new knowledge becomes available, practice guidelines are amended. Recently published material on the pathophysiology of acute respiratory distress in COVID-19 suggests that lung compliance is initially preserved, despite poor oxygenation. A ground glass appearance on computed tomography (CT) scans suggests interstitial rather than alveolar oedema. These patients are referred to as the ‘L-type’ patient (low elastance, high compliance). Some of these patients progress to the ‘H-type’ (high elastance, low compliance), which is the typical acute respiratory distress syndrome (ARDS) associated with a high mortality rate.

The South African clinical guidelines provide the following guidance:

Aim for an initial tidal volume of 4–6 ml/kg. Higher tidal volume up to 8 ml/kg predicted body weight may be needed if minute ventilation requirements are not met in a patient with good lung compliance. Strive to achieve the lowest plateau pressure possible. Plateau pressures above 30 cm H_2_O are associated with an increased risk of pulmonary injury.

An increased peak pressure and a difference of peak to plateau pressure of greater than 5 cm H_2_O usually implies airway resistance such as bronchospasm, secretions or mucus plugs. An increased plateau pressure and a smaller difference between peak and plateau pressures usually imply decreased compliance and this may be associated with acute respiratory distress syndrome, pneumonia or pneumothorax. In the case above, the tidal volume was initially set at 6 mL/kg, so it was acceptable to increase it to 8 mL/kg and continue to monitor the plateau pressures, as a rise in the plateau pressure may signify a loss in lung compliance and alternate strategies would then need to be considered. It is always prudent to consult an intensivist when decisions about ventilation are being made.

Acute respiratory distress syndrome is characterised by non-cardiogenic pulmonary oedema, hypoxaemia, and reduced aerated lung size and low lung compliance. In such circumstances, the aim is to increase lung size by recruiting previously collapsed alveoli by using higher levels of peak end-expiratory pressure and prone positioning. High transpulmonary pressures are poorly tolerated in acute respiratory distress syndrome, hence low tidal volumes with permissive hypercapnia help to prevent ventilator-induced lung injury. Strenuous spontaneous inspiratory efforts may also contribute to lung damage by increasing transpulmonary pressures, hence there may be a need to sedate the patient to prevent patient-induced lung injury.

Understanding lung physiology and the pathophysiology of SARS-CoV-2-induced lung injury is critical to ventilator use in the primary care setting. Family physicians may be called on to use these skills in the coming months. Resources to improve competencies are listed below.

- South African Department of Health and the National Institute of Communicable Diseases. Clinical management of suspected or confirmed COVID-19 disease [homepage on the Internet]. 3rd ed. Pretoria; 2020. Available from: https://www.nicd.ac.za/wp-content/uploads/2020/03/Clinical-Management-of-COVID-19-disease_Version-3_27March2020.pdf
- Marini JJ, Gattinoni L. Management of COVID-19 respiratory distress. JAMA. 2020. https://doi.org/10.1001/jama.2020.6825
- Seheult R. COVID-19 ventilator course: Learn or review mechanical ventilation [homepage on the Internet]. MedCram 2020. Available from: https://www.youtube.com/watch?v=mnIpD1VwyMo

## Short answer question: The family physician’s role as a care provider

You are working as a family physician in a rural district hospital. Your junior colleague has phoned you (as the senior on call for anaesthesia) to assist with a patient who presented to the emergency centre with severe acute respiratory infection. This is a 70-year-old male with a past medical history of hypertension and chronic obstructive lung disease, who has now presented with a 3-day history of sore throat, fever, worsening cough and shortness of breath. The patient’s brother (who lives in the same household) is being managed as a person under investigation for the coronavirus disease 2019 (COVID-19) and is awaiting his test result.

1. Describe the key clinical concerns to consider regarding this patient’s presentation. (5 marks)
2. You decide to prepare for intubation as the patient is becoming more hypoxic, confused and hypotensive. Which principles should you keep in mind for intubation and ventilation, with specific reference to the technical steps of the procedure as well as personal protective equipment? (5 marks)
3. As your junior colleague is helping to prepare the equipment described above, you contact your referral hospital. Discuss the ethical principles behind the South African triage guidelines used for all patients requiring intensive care during the COVID-19 pandemic. (2 marks)
4. With reference to the triage guidelines, the consultant from the referral hospital recommends a palliative approach for your patient. How would you communicate the decision of providing palliative care to the patient’s wife and son, who have just arrived? (The patient has become confused.) (4 marks)
5. How do you approach the clinical side of initiating palliative care, especially in terms of managing the distressing symptom of dyspnoea? (4 marks)

### Model answers

#### Describe the key clinical concerns to consider regarding this patient’s clinical presentation. (5 marks)

- This patient meets the case definition for COVID-19 and should be considered infectious. All necessary infection and prevention control measures should be implemented: The patient must wear a surgical mask and be evaluated in a single room or, ideally, the separate area of the emergency centre identified for emergency care of COVID-19 patients for urgent attention. Healthcare workers should wear personal protective equipment (PPE): contact and droplet precautions, as well as airborne precautions such as N95 respirator and eye protection. (This patient may need high-flow oxygen, nebuliser and possible intubation, which is an aerosol-generating procedure.) Limit patient movement. Rather use a portable x-ray machine if indicated.
- Ideally, a nasopharyngeal swab and, if present, a sputum sample, should be collected as specimens, to confirm infection with SARS-CoV-2. This public health responsibility (identifying an infectious patient via screening and testing, as well as tracing close contacts during the past 14 days), should be coordinated in a team-based manner with the person-centred care required by your patient (acute care support including respiratory support).
- Urgent attention should be given if your patient is showing signs of respiratory distress such as might be the case here. If the patient has a cough or difficulty breathing and any of the following: a respiratory rate ≥ 25 breaths per minute, becomes hypotensive (blood pressure < 90/60), pulse rate > 120 beats per minute, becomes confused or agitated and/or is unable to walk without help.
- Consider severe COVID-19 as well as other causes in your differential diagnosis and management plan, such as bacterial pneumonia, exacerbation of the patient’s underlying chronic obstructive lung disease, heart failure, a tension pneumothorax or a pulmonary embolus.
- On admission, it is important to start having the conversation around goals of care and identifying a healthcare proxy, especially given this patient’s age and co-morbidities. The Association of Palliative Care Practitioners of South Africa (PALPRAC) recommends that,
  [A]ll patients with underlying chronic illnesses and severe COVID symptoms should be considered for early supportive therapy (supplemental oxygen with or without empiric antimicrobials) as per the COVID Clinical Guidelines, unless resources do not allow for this or if the patient or their medical decision maker clearly states that they decline such therapy.

Clear guidelines have been provided by PALPRAC on pharmacological and non-pharmacological treatment options in their ‘Providing Palliative Care in South Africa during the COVID-19 Pandemic’ guidelines.

#### You decide to prepare for intubation as the patient is becoming more hypoxic, confused and hypotensive. Which principles should you keep in mind for intubation and ventilation, with specific reference to the technical steps of the procedure as well as personal protective equipment? (5 marks)

Here, it is essential to apply the recommended principles, such as those of the South African Association of Anaesthesiologists (SASA). The principles of managing this patient’s airway (a suspected COVID-19 case with progressive respiratory distress or failure) should be considered from three aspects, namely before, during and after intubation. During each step it is essential to remain mindful of the need to prevent staff contamination.

#### Before intubation

Start with focusing on staff protection: Ensure hand hygiene, full protective gear, minimise the number of staff members present during aerosol-generating procedures (as is the case of intubation), and aim to perform the intubation and ventilation in a dedicated area prepared for airborne infection isolation.

Preparation should now focus on the procedure itself: Ensure the early protection of drugs and equipment needed for intubation and emergency ventilation; perform airway assessment (anticipate a difficult airway); formulate a plan early in conjunction with the team in the emergency centre (and your referral institution); and, if time allows, use a closed suctioning system and connect the appropriate filter to the ventilator’s circuit and manual ventilator, such as an Artificial Manual Breathing Unit.

Whilst preparing, ensure that the patient has an intravenous access, receives oxygen via nasal oxygen and/or face mask, and receives other supportive treatment as needed (antibiotics, inotropes, diuretics, etc.). Remember to avoid giving nebulised treatment (an aerosol-generating procedure); rather give beta-agonists via an inhaler and spacer.

#### During intubation

Again, first focus on preparation: Ensure that there is clear delineation of roles (the SASA guidelines refer to ‘hot’ and ‘non-hot’ roles, to ensure minimise the number of staff exposed during this aerosol-generating procedures); other key aspects of team dynamics should include clear communication of the airway plan (such as escalation when encountering a difficult airway), closed loop communication throughout, and cross-monitoring by all team members for potential contamination.

Technical aspects to highlight, aimed at shortening the risk of staff contamination, include: performing airway management by the most experienced practitioner, use a tight-fitting mask with two hand grips to minimise leak, ensure paralysis to prevent coughing, using the lowest gas flows possible to maintain oxygenation, pre-oxygenating with 100% oxygen for 5 min, using rapid-sequence induction instead of prolonged bag-mask ventilation, and initiating positive pressure ventilation only after the endotracheal tube’s cuff is inflated.

#### After intubation

The need to prevent staff contamination should be ensured. Avoid unnecessary circuit disconnection (should this occur or be needed, wear PPE and consider clamping the endotracheal tube), maintain strict adherence to proper de-gowning (‘doffing’) steps and hand hygiene habits, and dispose of contaminated airway equipment appropriately. A team debriefing post-intubation is recommended, to review the treatment plan and support team members.

#### As your junior colleague is helping to prepare the equipment described above, you contact your referral hospital. Discuss the ethical principles behind the South African triage guidelines used for all patients requiring intensive care during the COVID-19 pandemic. (2 marks)

The Critical Care Society of Sourthern Africa (CCSSA) provides guidance on triaging scare resources. The ethical guidelines of the South African Medical Association – SAMA (https://www.samedical.org/cmsuploader/viewArticle/1139) – also refer to the CCSSA triage guidance.

In the introduction to the document, the following text is provided (a model answer will capture these key elements):

The purpose of the CCSSA guideline is to provide guidance for the triage of critically ill patients in the event that a public health emergency creates demand for critical care resources (e.g. ventilators, critical care beds) that outstrips the supply.

These triage recommendations will be enacted only if:

- critical care capacity is, or will shortly be, overwhelmed despite taking all appropriate steps to increase the surge capacity to care for critically ill patients; and
- His Excellency, the President of South Africa, has declared a public health emergency.

This allocation framework is grounded in ethical obligations that include:

- duty to care,
- duty to steward resources to optimise population health,
- distributive and procedural justice, and
- transparency.

It is consistent with existing recommendations for how to allocate scarce critical care resources during a public health emergency.

#### With reference to the triage guidelines, the consultant from the referral hospital recommends a palliative approach for your patient. How would you communicate the decision of providing palliative care to the patient’s wife and son, who have just arrived? (The patient has become confused.) (4 marks)

The PALPRAC guidelines describe approaches to various scenarios one may encounter during the COVID-19 pandemic and describe key aspects of communication in these scenarios. The guidelines highlight the unique challenges presented by the COVID-19 pandemic, especially the issues of staff safety and the potential absence of family at the bedside. Once the decision has been made by the clinical team to withdraw or withhold ventilatory support, this decision should be documented in the clinical notes. Guidelines for family communication are provided and this conversation should also be captured in the clinical notes. Skilled and compassionate communication should include the following steps from page 15 of the PALPRAC guidelines (a model answer will include elements of these steps):

Always start by checking the patient/family member’s understanding of the situation and ask what they have been told before. There are often clues for you to use in order to take the conversation forward.

- Give information in small, digestible chunks, avoiding medical jargon.
- Use silence – this allows people to absorb what was said and show emotion.
- Acknowledge emotion: NURSE acronym
  ■ Name emotion: ‘You seem to be upset/worried?’
  ■ Understanding: ‘Given what is going on, I can understand your concern’.
  ■ Respecting: ‘You have been really patient under difficult circumstances’.
  ■ Supporting: ‘I understand that this is very hard. We will be here to help’.
  ■ Exploring: ‘Tell me more, I would like to understand what you’re thinking’.
- Never say: ‘There is nothing more that we can do for you/your mother…’. Commit to excellent symptom management, compassionate communication and your presence.
- Consider linking family telephonically or online to say a final goodbye.
- Consider arranging a tablet or phone in a wipe-able pouch for the unit for WhatsApp video calls or equivalent.

#### How do you approach the clinical side of initiating palliative care, especially in terms of managing the distressing symptom of dyspnoea? (4 marks)

A model answer will include aspects of the proposed management for ‘Hospital-based care for patients with severe symptoms who are not candidates for critical care admission & ventilation if they deteriorate’ (see pages 8–12 of the PALPRAC guidelines; these and other resources are available from: https://palprac.org/for-healthcare-providers/palliativecarecovid-19/).

Oxygen therapy is likely to be the single most effective supportive measure in COVID-19 patients overall. Any patient with hypoxaemia (saturation < 90%) should be given supplemental oxygen to achieve O_2_ saturation > 90%.

Non-pharmacological interventions should be focused on addressing restlessness (nursing care, mouth care, keeping the patient comfortable) and shortness of breath (positioning, breathing exercises, emotional and spiritual care).

Pharmacological interventions are determined by the grade of dyspnoea (see page 9 of the PALPRAC guidelines) and may include morphine (orally or subcutaneously/intravenously) and anxiolytics or sedatives such as lorazepam or midazolam which may be provided via an ambulatory syringe pump (syringe driver) to deliver a continuous subcutaneous infusion. Morphine, metoclopramide and midazolam may be combined and administered via continuous subcutaneous infusion or via an intravenous infusion pump. Reassess and adjust rate if the patient is not comfortable or give additional breakthrough doses (2.5 mg morphine and 2.5 mg – 5 mg midazolam stat subcutaneously or intravenously). In the elderly and those with renal failure, start at lower doses. An alternative to morphine includes fentanyl (if available). Should no syringe driver be available and the patient is unable to swallow, the subcutaneous bolus administration route may be used.

**FIGURE 1:**
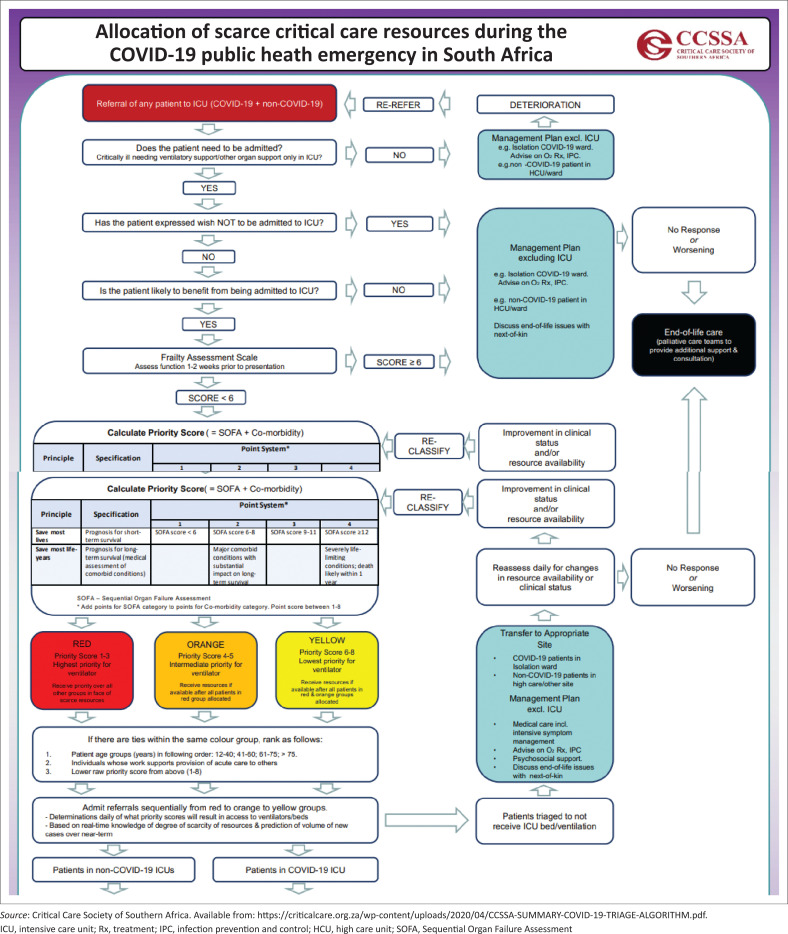
Excerpt of algorithm: Allocation of scarce critical care resources during the COVID-19 public health emergency in South Africa.

Further reading:

- COVID-19 guidelines [homepage on the Internet]. National Institute for Communicable Diseases; 2020 [cited 2020 May 2]. Available from: https://www.nicd.ac.za/diseases-a-z-index/covid-19/
- SASA COVID-19 update [homepage on the Internet]. South African Society of Anaesthesiologists; 2020 [cited 2020 May 2]. Available from: https://sasacovid19.com/
- COVID-19 airway management resources [homepage on the Internet]. OpenAirway; 2020 [cited 2020 May 2]. Available from: https://openairway.org/covid-19-airway/
- Providing palliative care in South Africa during the COVID-19 pandemic [homepage on the Internet]. The Association of Palliative Care Practitioners of South Africa; 2020 [cited 2020 May 2]. Available from: https://palprac.org/
- Mash B. Primary care management of the coronavirus (COVID-19). S Afr Fam Pract [serial online]. 2020 [cited 2020 May 2]. Available from: https://doi.org/10.4102/safp.v62i1.5115
- Coronavirus (COVID-19) and other resources [homepage on the Internet]. Knowledge Translation Unit, University of Cape Town Lung Institute. [cited 2020 May 2]. Available from: https://knowledgetranslation.co.za/resources/
- Allocation of scarce critical care resources during the COVID-19 public health emergency in South Africa [homepage on the Internet]. Critical Care Society of Southern Africa [online]. 2020 [cited 2020 May 2]. Available from: https://criticalcare.org.za/covid-9/
- SARS-CoV-2 (COVID-19) guidance for managing ethical issues [homepage on the Internet]. South African Medical Association. [cited 2020 May 2]. Available from: https://www.samedical.org/cmsuploader/viewArticle/1139
- Coronavirus disease (COVID-19) pandemic [homepage on the Internet]. World Health Organization. 2020 [cited 2020 May 2]. Available from: https://www.who.int/emergencies/diseases/novel-coronavirus-2019
- COVID-19 corona virus South African resource portal [homepage on the Internet]. Pretoria: National Department of Health; 2020 [cited 2020 May 2]. Available from: https://sacoronavirus.co.za/

## Critical appraisal of quantitative research

Read the accompanying article carefully and then answer the following questions (*total 32 marks*). As far as possible use your own words. Do not copy large portions of the article. Be guided by the allocation of marks with respect to the length of your responses.

Alabi AA, Adeniyi OV, Adeleke OA, Pillay P, Haffajee MR. Factors associated with failed spinal anaesthesia for Caesarean sections in Mthatha general hospital, Eastern Cape, South Africa. S Afr Fam Pract [serial online]. 2017;59(4):128–132. https://doi.org/10.1080/20786190.2017.1292696

1. Comment on the scientific and social value of this study (2 marks)
2. What were the aims of the study? (1 mark)
3. Comment critically on the dates of the references as well as the appropriateness of references 1 and 2. (3 marks)
4. Comment critically on the study population. (5 marks)
5. What was/were the definitions of a failed spinal? (3 marks)
6. In light of the limitations mentioned, would you have confidence that the definitions were consistently applied? Substantiate your answer. (2 marks)
7. From Table 2, can you work out how many women needed a general anaesthetic? Substantiate your answer. (2 marks)
8. Calculate and comment on the risk ratio of a failed spinal anaesthetic for an emergency caesarian section versus an elective caesarian section. (3 marks)
9. Were there any significant complications presented which were not discussed in this article? (4 marks)
10. In the discussion, the authors comment on the association between a bloody tap and a failed spinal (see extract below).
  Bloody CSF from an initial attempt was significantly associated with FSA in our study. This finding has significant clinical and academic implications; there is a greater likelihood of inaccurate placement of the spinal needle into a vessel and, hence, higher FSA and other complications following intravascular injection of bupivacaine.

Comment critically on both the clinical and academic implications to which the authors allude. (2 marks)

11. Do the data presented in Table 3 support the conclusions that anaesthetic training amongst junior doctors should be prioritised? (2 marks)
12. What is your take-home message from this article? (3 marks)

### Model answers

#### Comment on the scientific and social value of this study.

**Scientific value:** In the face of increasing numbers of caesarian section (CS) deliveries, little is known about the incidence and factors contributing to failed spinal anaesthesia (FSA) amongst pregnant women going for CS in the Eastern Cape. It is important to understand the factors contributing to FSA so that these can be addressed.

**Social value:** Caesarian section is a common procedure and it important to understand factors contributing to failed spinal anaesthesia so as to be able to offer a safe service to those needing a CS. Value also lies in patient-centred counselling regarding FSA, at a time when patients have more knowledge or curiosity about their medical care. ([2] 1 mark for each)

#### What were the aims of the study?

**Aims:** Determine the incidence of failed spinal anaesthetic in pregnant women presenting for CS delivery AND identify the contributory factors to the failure of spinal anaesthesia in Mthatha General Hospital. (1 mark)

#### Comment critically on the dates of the references as well as the appropriateness of references 1 and 2.

18/25 (64%) of the references are more than 5 years old. Ideally > 80% – 85% of references in a manuscript should be less than 5 years old.

**Reference 1** is the reference given for increasing CS rate globally and spinal anaesthetic as the anaesthetic of choice for this procedure. However, reference 1 (Páez JJ, Navarro JR. Anestesia regional versus general para partopor cesárea. Rev Colomb Anestesiol. 2012;40:203–206) is not a meta-analysis of global trends or a World Health Organization report on global trends, but an article in Spanish from Colombia which does not seem to be an appropriate reference. (The abstract from the article states: ‘*Methods:* Article for reflection. *A non-systematic search* of the literature on the topic was performed in the Medline/Pubmed, Embase, Cochrane and Lilacs databases, using the following Mesh terms: Cesaerean section, General anesthesia, Spinal anesthesia, Epidural anesthesia. *Results:* Although the *rates for cesarean sections have been constant*, the use of general anesthesia has decreased progressively. Maternal mortality associated to general anesthesia during cesarean section has dropped to practically the same level as regional anesthesia: 1.7 (95% confidence interval [CI]: 0.6–4.6). Mortality is lower with regional anesthesia: less bleeding, lower risk of surgical site infection, less post-operative pain. The neonatal outcomes are practically the same.’) The abstract contradicts the statement, which is that there is an increasing CS rate globally.

**Reference 2** is a thesis from the University of the Witwatersrand which is not an appropriate *primary* source of data supporting the increasing rate of CS delivery in South Africa. Although it is ‘a comprehensive analysis of C-section rates in all public sector hospitals during 2000/2001–2008/2009 by facility, district and province’, it uses ‘secondary data obtained from the National Department of Health’s routinely collected data specific to Caesarean sections in the DHIS’. A better source would have been the confidential enquiry into maternal deaths, mentioned later, or National Department of Health figures. ([3] 1 mark for comments about the dates of references and 1 mark for comments about references 1 and 2)

#### Comment critically on the study site and study population.

No details are given about the services provided at Mthatha General Hospital in terms of the area served, the number of deliveries and the number of CSs’ done. This makes it difficult to place the study within the context of the services provided and makes it difficult to apply the findings to other (similar) contexts. (2 marks)

The abstract states that there were 197 pregnant women scheduled for CS included in the study. In the manuscript it states that there were 200 consecutive pregnant women enrolled in the study. In the results section it states that 197 participants were included in the final analysis; incomplete data for three were excluded – suggesting a sample of 194. Data are presented on 197. (1 mark)

No details are given of the total study population and why a number of 200 was chosen and whether or not this was considered representative of those undergoing caesarean deliveries. The authors state that 200 consecutive pregnant women were enrolled, suggesting that this is the TOTAL population and not a sample from the total population, but this is not stated. (1 mark)

In addition, the data were collected in 2013 and only published in 2017, making the data old and out of date. (1 mark)

#### What was/were the definitions of a failed spinal? (3 marks)

- Women experiencing pain after 10 min of administering spinal anaesthesia.
- Women who experienced pain during the procedure whose pain persisted after being given pethidine.
- Women without any sensory block (see discussion).

#### In light of the limitations mentioned, would you have confidence that the definitions were consistently applied? Substantiate your answer. (2 marks)

The limitations state that: (1) the time of failure of spinal anaesthesia was not documented in the study and (2) it is possible that a number of spinals were repeated as they state ‘We also do not have data on the number of FSAs which were repeated’. (Surely this should have been recorded in the notes if comprehensive records are being kept, as suggested by the quantity of data being collected.)

It is by no means clear that the definitions were consistently applied. If no one has recorded the time the anaesthetic started, it makes it difficult to know whether 10 min was used to assess whether or not the spinal anaesthetic had failed or not.

In addition, as no record of whether or not an additional spinal was given if the initial spinal failed, it is impossible to know whether or not the findings (and complications) are based on one or more attempts and what the implications of repeat spinals are.

#### From Table 2, can you work out how many women needed a general anaesthetic? Substantiate your answer. (2 marks)

No – Table 2 needs to be read in conjunction with the discussion if one is to work out how many women needed a general anaesthetic.

The discussion states: A failure rate of 14.2% (of 169 = 24 patients) and 10.5% (of 21 = 2 patients) was observed with use of L3–L4 and L4–L5. Total of 26 patients, not 23 patients, with failed spinal anaesthetic.

Patients who achieved a block height of T8–T10 had 100% failure rate (3) while 33.3% (of 27 = 9 patients) and 1.3% (of 158 = 2 patients) failure rates were seen at block heights T6–T7 and T4–T5, respectively. Total of 14 patients plus 9 with not block makes 23 patients with failed spinal anaesthetic.

Results should be presented in the results section and should be able to stand alone. The discussion should discuss the results and not provide additional results.

#### Calculate and comment on the risk ratio of a failed spinal anaesthetic for an emergency caesarian section versus an elective caesarian section. (3 marks)

This suggests that there is a 1.4 times greater chance of having a failed spinal during an emergency CS than during an elective CS. However, the 95% confidence crosses ‘1’, which makes this not significant, and the *p*-value is high, which also indicates that this is not a significant finding. (Table 1) (1 mark)

#### Were there any significant complications presented which were not discussed in this article? (4 marks)

The authors state that a significant number of women developed complications – hypotension 39%, shivering 16%, vomiting 6.9%, mortality 4.3%, pain 2.9% and headache 2.9%. (1 mark)

While the focus of the article is on failed spinal and the reasons for this, significant adverse events such as hypotension and shivering, as well as a *mortality rate* of 4.3% (9/197 patients died) are very significant and should have been commented on and discussed. Shivering is touched on but no mention is made of the hypotension or the mortality. In addition, it is difficult to understand the reported pain of 2.9%. (2 marks)

Is this women who experienced pain during the procedure (they should have been converted to a general anaesthesia) or is this pain at the injection site? In what way would this be considered a complication of spinal anaesthesia? (1 mark)

#### In the discussion, the authors comment on the association between a bloody tap and a failed spinal (see extract below).

Bloody CSF from an initial attempt was significantly associated with FSA in our study. This finding has significant clinical and academic implications; there is a greater likelihood of inaccurate placement of the spinal needle into a vessel and, hence, higher FSA and other complications following intravascular injection of bupivacaine.

#### Comment critically on both the clinical and academic implications to which they allude. (2 marks)

It is difficult to understand what the authors are referring to in this paragraph and what the academic and clinical implications are. No clear definition is given about what would be considered to be a bloody tap. The understanding of a bloody tap is that there is a small amount of blood in the CSF as it flows back through the spinal needle, suggesting that one is in the correct space but has injured a vessel when doing the spinal. Usually, one would allow the blood to clear and then inject the bupivacaine. If it does not clear but blood continues to flow, one would conclude that one is in a vessel and would remove the spinal needle and NOT inject the bupivacaine. In the article, there is no evidence from the study or from the literature of complications following intravascular injection of bupivacaine.

**TABLE 1:** Part of model answers - 2 × 2 table showing failed and successful spinal anaesthetics.

Risk ratios	Emergency CS		Elective CS		Totals
	*n*	%	*n*	%	
Failed spinal anaesthetic	19	12.3	4	9.3	23
Successful spinal anaesthetic	135	87.7	39	90.7	174
**Totals**	**154**	**-**	**43**	**-**	**197**

CS, caesarian section.

Numbers in bold are calculated totals (1 mark).

Risk ratio: (19/135)/(4/39) = 0.141/0.103 = 1.37 (95% CI: 0.48 – 3.69, *p* = 0.59). (1 mark)

The academic and clinical implications may be the need to correctly teach students and clinicians how to do a spinal anaesthetic using appropriate landmarks and to understand the implications of a bloody tap – when experiencing a bloody tap, to allow the blood to clear prior to injecting bupivacaine and to ensure free flow of CSF, and not to the inject bupivacaine if there is only blood draining from the CSF needle, but rather to remove the spinal needle and redo the spinal.

#### Do the data presented in Table 3 support the conclusions that anaesthetic training amongst junior doctors should be prioritised? (2 marks)

In Table 3, years of experience is reported as being significantly associated with the increased chance of a failed spinal and seems to support the conclusion that anaesthetic training amongst junior doctors should be prioritised. However, although this may be true, no details are given regarding the experience of those giving the anaesthetic other than to mention how many of the 197 anaesthetics were given by each category of staff (FP – 19, registrars – 80, medical officers – 90, community service officers – 8) and experience is probably a more important indicator than years of service. It is also unfortunate that this was presented as a binary < or > 1 year, as there might also be a falloff in competencies of more senior staff who are no longer routinely involved in giving anaesthetics.

**FIGURE 2:**
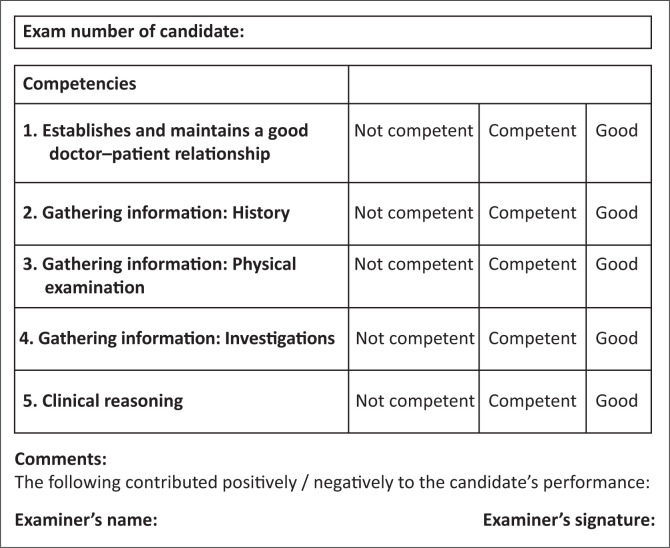
Marking template for consultation station.

#### What is your take-home message from this article? (3 marks)

**Relevance:** The research is relevant to the generalist family physician working at a district hospital.

**Education:** It has added to the knowledge of the factors associated with failed spinal anaesthetics.

**Applicability:** The research could have been done in a similar setting.

**Discrimination:** There are concerns about the sample size and selection, how the data were collected, some of the limitations and how these impact the results, the quality of the service provided (as reflected in the high mortality rate).

**Evaluation and reaction:** There is nothing that one would take from this article to apply to one’s own practice. (1/2 mark/s for something in each section)

With thanks to Dr A. Asghar and Dr R. Ali for their input.

## Objectively Structured Clinical Examination scenario

### 

#### Objectives of station

This station tests the candidate’s ability to:

- Conduct a pre-operative consultation for an elective caesarean section, including a risk assessment.
- Manage a high-risk patient appropriately.

#### Type of station

Integrated consultation.

#### Equipment list

- Simulated patient: 26-year-old pregnant patient (37 weeks’ gestation).
- Torch light to visualise oral cavity.
- Stethoscope.
- Chest x-ray of a mitral stenosis patient.
- Electrocardiogram of a mitral stenosis patient.

## Instructions for candidate

### 

#### History/context

You are the family physician working in a rural district hospital. A 26-year-old woman with a history of shortness of breath for 1 week, at 37 weeks of gestation, was reviewed in the antenatal clinic and referred to you. The attending clinician commented, ‘high risk pregnancy with cardiac condition’ and advised to book for elective caesarean section. The patient’s basic workup has been done.

#### Your task

- Do a pre-operative anaesthetic fitness consultation.
- Negotiate a management plan appropriate to your assessment.

N.B.: You do not need to do an examination on this patient. All examination findings will be provided on request.

## Instructions for the examiner

### 

#### Objectives

This station tests the candidate’s ability to:

- Conduct a pre-operative consultation for an elective caesarean section, including a risk assessment.
- Manage a high-risk patient appropriately.

**FIGURE 3:**
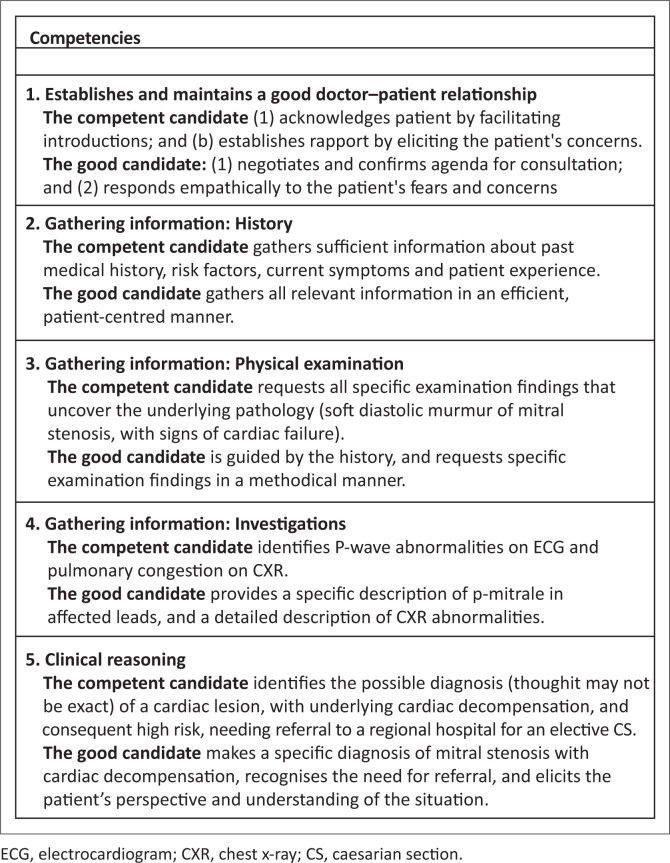
Guidelines for examiners to judge student competencies.

This is an integrated consultation station in which the candidate has 14 min. Familiarise yourself with the Assessor guidelines (Figure 3) which detail the required responses expected from the candidate.

No marks are allocated. In the mark sheet, tick off one of the three responses for each of the competencies listed. Make sure you are clear on what the criteria are for judging a candidate’s competence in each area. Provide the following information to the candidate when requested:

#### Examination findings

Electrocardiogram and chest x-ray – candidate to interpret.

Please switch off your cell phone.

Please do not prompt the student.

Please ensure that the station remains tidy and is reset between candidates.

This station is 15 min long. The candidate has 14 min, then you have 1 min between candidates to complete the mark sheet and prepare the station.

**Reference:** Klocke M. How to do a pre-anaesthetic consultation. In: Mash B, Blitz J, editors. SA family practice manual. 3rd ed. Pretoria: Van Schaik, 2015; pp. 422–425.

## Guidance for assessors

Figure 2 are relevant clinical competencies extracted for the OCSE station from the mini CEX (mini clinical evaluation exercise). This is a validated examination tool that is used in the registrars portfolio of learning. The aim is to establish that the candidate is able to safely and effectively identify that this patient is too high a risk for a district hospital and needs to be transferred to a regional hospital.

Competent: The task is completed safely and effectively.

Good: In addition to displaying competence, the task is completed efficiently and in an empathic, patient-centred manner (acknowledges the patient’s ideas, beliefs, expectations, concerns and fears).

### Role play – Instructions for actor

#### Appearance (including dress) and behaviour (emotions and actions)

Pregnant woman in her 20s’. Some difficulty breathing with minimal activity. Anxious about the condition and future of own and baby’s health.

**Opening statement:** (Responds to doctor) ‘Dr, I’m worried – why did the other doctor say I must come see you. Is the baby OK?’

### History

#### 

##### Open responses: Freely tell the doctor if asked …

This is your first pregnancy. Everything was fine at all your check-ups so far.

In the last 3 weeks, you developed swelling in your legs. At first you thought it is normal to be tired and for your legs to get swollen in pregnancy. But now it seems that it is getting worse. Since last week, you also developed difficulty breathing.

##### Closed responses: Only tell the doctor if s/he brings this up

You know that the other doctor in maternity suspects that you might have a heart condition. No one in your family has ever had anything like this.

You planned this pregnancy. You are married, and your husband is at work.

You don’t drink or smoke. You have never really been involved in exercise or sports. You don’t have any medical problems, and apart from the vitamins for pregnancy, you don’t take any other medications.

You have never been in hospital before, and you work as a receptionist at a local dentist.

##### Ideas, concerns and expectations

You are worried that your baby may be affected, and that you will have a long-term problem. What does all this mean?

The doctor must do whatever must be done to ensure that the baby will be fine.

#### Patient’s notes/examination findings

**FIGURE 4:**
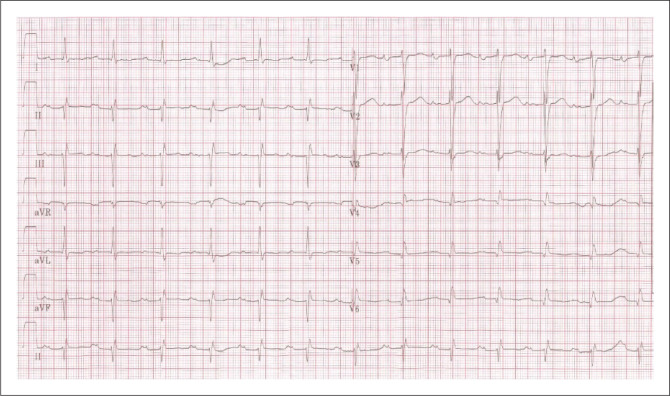
Electrocardiogram (ECG) of patient.

**FIGURE 5:**
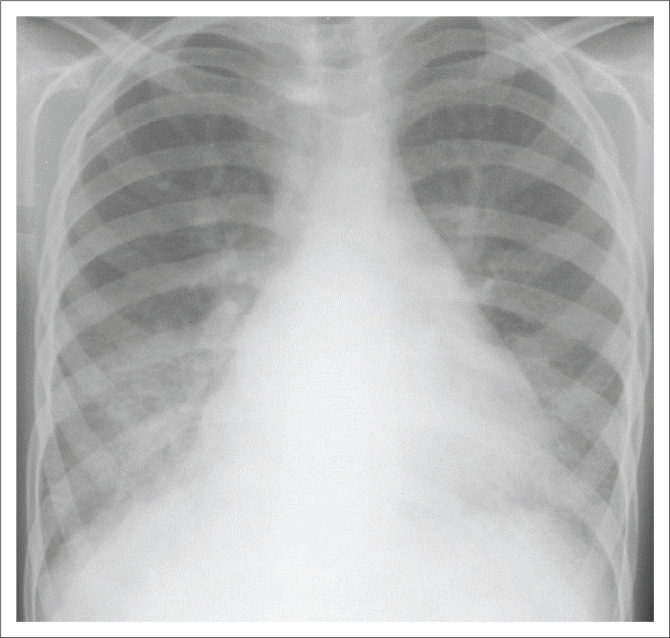
Chest x-ray of the patient.

- Body mass 72 kg
- Height 1.57 m
- BP 130/80 mm-Hg
- Hb 9.4 gm/dL
- Serum creatinine 85 *µ*mol/L (ref 49–90)
- Urea 6.5 mmol/L (ref < 8.4)
- Random blood glucose (HGT) 8 mmol/L
- Urinalysis: Protein (trace)
- Shallow tachypnoea with respiratory rate of 23/min
- Bilateral pedal oedema (++)
- Chest: Crackles at both lung bases
- CVS: Heart rate 96/min
  ■ Diastolic murmur of low pitch, rumbling in character at the apex
- Chest x-ray: Enlarged heart shadow (Figure 5)
  ■ Straightening of left cardiac border
  ■ Prominent pulmonary vessels
  ■ Presence of Kerley B lines
- ECG: Bifid P-waves (wide > 0.11 s) in Lead I, II and Avl. (Figure 4)

## Data Availability

Data sharing not applicable - no new data was generated.

